# From Forestry By-Product to Functional Food Ingredient Innovation: Antiproliferative, Antimetastatic and Antiplasmodial Activities of Norway Spruce Sawdust Extract

**DOI:** 10.3390/foods15020264

**Published:** 2026-01-11

**Authors:** Julia Carvalho Cardoso Consentini, Gabriela Furlaneto, Nathália Alves Bento, Thaise Caputo Silva, Fernando Vitor Vieira, Petri Kilpelainen, Giselly Karoline Paiva da Silva, Ignasi Bofill Verdaguer, Marcell Crispim, Amanda dos Santos Lima, Luciana Azevedo

**Affiliations:** 1 Laboratory of Nutritional and Toxicology Analysis In Vitro and In Vivo, Federal University of Alfenas, Alfenas 37133-840, MG, Brazil; julia.consentini@sou.unifal-mg.edu.br (J.C.C.C.); gabriela.furlaneto@sou.unifal-mg.edu.br (G.F.); nathalia.bento@sou.unifal-mg.edu.br (N.A.B.); thaise.caputo@sou.unifal-mg.edu.br (T.C.S.); fernando.vieira@sou.unifal-mg.edu.br (F.V.V.); giselly.silva@sou.unifal-mg.edu.br (G.K.P.d.S.); 2 Natural Resources Institute Finland (Luke), Latokartanonkaari 9, FI-00790 Helsinki, Finland; petri.kilpelainen@luke.fi; 3 Department of Parasitology, Institute of Biomedical Sciences, University of São Paulo, São Paulo 05508-000, SP, Brazil; ignasibofill@usp.br; 4 Department of Clinical and Toxicological Analysis, School of Pharmaceutical Sciences, Federal University of Alfenas, Alfenas 37130-001, MG, Brazil; marcell.crispim@isglobal.org; 5 Barcelona Institute for Global Health (ISGlobal), Hospital Clínic-Universitat de Barcelona, 08036 Barcelona, Spain

**Keywords:** Norway spruce, phenolic compounds, antiproliferative activity, antiplasmodial activity, *Plasmodium falciparum*, malaria

## Abstract

The Norway spruce (*Picea abies*) is a forest resource whose by-products contain bioactive compounds such as galactoglucomannan (GGM), catechin, and epicatechin, recognized for their antioxidant and chemopreventive potential. Within a food-related valorization context, we evaluated the antiproliferative, antimetastatic, genotoxic, and antimalarial activities of the Norway spruce by-product extract (NSBE). Considering its chemical composition and multifunctional bioactive profile, NSBE is investigated for its potential application as a functional food ingredient. NSBE exhibited concentration-dependent antiproliferative and antimetastatic effects two cancer cell lines (A549 and HCT-8), reducing cell adhesion by 33.96% in A549 cells and 40.15% in HCT-8 cells, and suppressing clonogenic capacity by up to 90% and 75%, respectively. The extract preserved basal chromosomal integrity and demonstrated a cytoprotective effect at 10 µg GAE/mL, reducing cisplatin-induced genotoxicity. Additionally, in antiplasmodial assays, NSBE showed potent inhibition of two *Plasmodium falciparum* strains: W2 (chloroquine-resistant) and 3D7 (chloroquine-sensitive) strains, with IC_50_ values below 3.5 µg GAE/mL. This activity was supported by a selectivity index (SI) of 13, exceeding the recommended threshold for natural antimalarial candidates. Altogether, these findings highlight the NSBE as a sustainable and multifunctional food ingredient with relevant antiproliferative and antiplasmodial properties, supporting its cytoprotective and chemopreventive potential within a functional food framework.

## 1. Introduction

The sawdust from coniferous trees is considered a forest industrial by-product and is usually discarded or combusted to generate energy, leaving a large amount of residue that is not fully utilized. The composition of this lignocellulosic biomass includes bioactive polysaccharides, notably hemicelluloses, along with polyphenolic compounds, which have been widely recognized for their health-related properties and their applicability in technological and functional developments within the food and pharmaceutical industries [1]. Consistent with the 2030 Agenda for Sustainable Development, the United Nations, which emphasizes sustainable consumption and production practices, the valorization of lignocellulosic residues through their integration into biorefinery processes represents a strategic approach toward a more circular and sustainable bioeconomy [2]. Within this framework, such residues, abundant yet underutilized, emerge as promising matrices for the extraction of functional and potentially therapeutic ingredients [3].

Studies have explored plant by-product extracts such as jabuticaba tree leaf [4]; camu-camu seed [5]; *Tapirira* seed extract [6] and reported promising antioxidant, anti-inflammatory, and antimalarial properties. In line with this growing trend, we investigated the conifer *Picea abies* (Norway spruce), a species whose by-products represent a valuable source of bioactive polysaccharides and phenolic compounds. The Norway spruce by-product extract (NSBE) is known to contain a galactoglucomannan-rich matrix associated with phenolic constituents such as catechin and epicatechin [7]. The coexistence of these flavanols within the polysaccharide framework has been reported to promote synergistic biological effects, enhancing antioxidant, anti-inflammatory, and immunomodulatory responses [7]. From an applied perspective, the chemical composition of NSBE highlights its potential as a multifunctional ingredient derived from renewable forest resources. The combined presence of galactoglucomannan (GGM) and phenolic compounds not only supports its technological functionality but also confers biological properties that may be exploited in the development of health-promoting products, including functional foods aimed at reducing disease risk through preventive strategies [8]. Additionally, GGM has been proposed as a multifunctional food ingredient due to its ability to stabilize delivery systems and protect bioactive compounds during gastrointestinal digestion, thereby potentially enhancing their bioaccessibility. Moreover, its resistance to digestion suggests a possible prebiotic role, as it may serve as a substrate for the intestinal microbiota [1].

Phenolic compounds, such as those present in the NSBE extract, are recognized as functional food ingredients, primarily because of their antioxidant capacity, which plays a crucial role in protecting the organism against oxidative stress [8]. Oxidative stress arises from an imbalance between reactive oxygen species and antioxidant defenses; this condition has been closely linked to the development of non-communicable diseases, including cardiovascular disorders, cancer, and neurodegenerative conditions [9]. Beyond their antioxidant role, phenolic compounds have demonstrated cytotoxicity toward cancer cells [10], anti-inflammatory [11], antimicrobial and anti-hypertensive [12], in addition to anti-hemolytic and anti-hyperglycemic activities [13]. In this context, the phenolic compounds identified in the NSBE, particularly catechin and epicatechin, may be involved in the regulation of redox imbalance and inflammatory signaling pathways associated with cancer progression and metabolic regulation.

In addition to these effects, phenolic compounds have also been reported to exert antiparasitic activity, particularly against *Plasmodium falciparum*, the etiological agent of malaria. This parasitic infection remains a significant global public health concern that disproportionately affects tropical and subtropical regions, where it is responsible for an estimated 600,000 deaths annually, with the highest incidence occurring across sub-Saharan Africa [14]. The rise in *Plasmodium falciparum* strains with reduced susceptibility to conventional antimalarial therapies has significantly compromised treatment efficacy, underscoring the urgent need to identify novel bioactive molecules from natural sources with potential therapeutic relevance. Although the antimalarial activity of Norway spruce phenolics has not yet been described, evidence from other plant sources demonstrates that similar compounds, such as epicatechin, catechin, quercetin, and gallic acid, can inhibit or reduce *Plasmodium* growth in vitro [5,15].

Despite promising evidence regarding the composition and bioactivity of NSBE, a comprehensive understanding of its toxicological safety, biological activity, and functional potential remains essential, with particular emphasis on cytotoxicity, genotoxicity, chromosomal integrity, and selectivity toward cancerous versus non-cancerous cells. This aspect is especially relevant as it represents an extract from an unconventional source intended for applications in food and pharmaceutical formulations derived from renewable resources [16]. From a toxicological standpoint, ensuring the absence of cytotoxic, genotoxic, and mutagenic effects is a fundamental prerequisite for validating novel bioactive ingredients and supporting their safe use in human-related applications. Despite existing evidence on NSBE composition and general bioactivity, studies integrating metastasis-related cancer phenotypes, chromosomal safety/genoprotection, and antiplasmodial activity within a framework of functional food ingredient valorization remain scarce. Consequently, exploring the biological mechanisms underlying NSBE activity is crucial for assessing its suitability as a safe and functional bioingredient.

Therefore, this study aimed to explore the biological effects of a Norway spruce by-product extract (NSBE) as part of an innovative valorization strategy for its potential application as a functional food ingredient. To this end, in vitro assays were conducted in lung adenocarcinoma epithelial cells (A549) and human ileocecal adenocarcinoma cells (HCT-8) to assess its effects on cell viability, adhesion, migration, and colony formation, as well as its genotoxic and antimalarial potential, to elucidate its cytoprotective and chemopreventive properties within a food-related context.

## 2. Materials and Methods

### 2.1. Chemicals and Reagents

For the analysis of phenolic composition, analytical standards including quercetin, chlorogenic acid, (+)-catechin, and gallic acid, purchased from Sigma-Aldrich (São Paulo, SP, Brazil), were used for compound identification and quantification. Reagents used in the spectrophotometric assays included ferric chloride hexahydrate and sodium nitrite (Sigma-Aldrich, São Paulo, SP, Brazil); sodium molybdate (Reatec, Colombo, PR, Brazil); hydrochloric acid (37%) and sulfuric acid (98%) (Fmaia, Indaiatuba, SP, Brazil); anhydrous sodium acetate (Anidrol, São Paulo, SP, Brazil); as well as vanillin, sodium hydroxide, ethanol, and aluminum chloride hexahydrate (Dinâmica, Indaiatuba, SP, Brazil). For cell-based assays, 3-(4,5-dimethylthiazol-2-yl)-2,5-diphenyltetrazolium bromide (MTT) was used to evaluate cell viability. Dulbecco’s Modified Eagle’s Medium/Nutrient Mixture F-12 Ham (DMEM/F-12), penicillin–streptomycin, and disodium ethylenediaminetetraacetate dihydrate (EDTA) were employed for cell culture and maintenance. Cisplatin was used as a positive control in the chromosomal aberration assay to induce chromosomal damage. All reagents were purchased from Sigma-Aldrich (São Paulo, SP, Brazil). For antimalarial assays, fluorescence-based parasite quantification was performed using SYBR Gold nucleic acid stain (S11494; Sigma-Aldrich, São Paulo, SP, Brazil). To maintain in vitro parasite cultures, Roswell Park Memorial Institute (RPMI)-1640 medium supplemented with Albumax I was used, both obtained from Gibco (New York, NY, USA).

### 2.2. NSBE Phenolic Composition

The Norway spruce (*Picea abies*) sawdust used for NSBE production was obtained as an industrial by-product, according to published studies using the same batch of raw material and extraction platform [3,17]. The sawdust consisted of non-sieved or fractionated particles with a broad size distribution, predominantly in the range of approximately 0.2–4 mm. No selective removal of bark fragments was performed prior to extraction. Moisture content of the raw sawdust was controlled during processing and storage as part of the industrial handling protocol. To obtain the NSBE, pressurized hot water extraction (PHWE) [17] was employed, a green and scalable technology that selectively solubilizes hemicelluloses from sawdust under elevated temperature and pressure [18]. Briefly, extraction was carried out in a flow-through system at 170 °C for approximately 60 min [17]. The resulting extract was subsequently concentrated by ultrafiltration using modified polyethersulfone membranes under near-neutral pH conditions, and the concentrate was spray-dried to obtain a GGM-rich product [3,17].

Following extract production, its phenolic composition was characterized using complementary colorimetric assays targeting different phenolic subclasses. The ortho-diphenol content was assessed by forming a colored complex between the ortho-dihydroxyl phenolic groups and sodium molybdate, resulting in a characteristic change in absorbance measured spectrophotometrically at 370 nm [19]. Quantification was conducted using a chlorogenic acid calibration curve, and results are expressed as milligrams of chlorogenic acid equivalents per gram of NSBE (mg CAE/g). To further evaluate condensed phenolic structures, tannin content was determined using the vanillin–HCl method, in which condensed tannins react with vanillin under acidic conditions to produce a red-colored chromophore, whose absorbance was measured at 500 nm after 15 min of incubation at room temperature [20]. Quantification was based on a (+)-catechin calibration curve, and values are expressed as milligrams of catechin equivalents per gram of NSBE (mg CE/g). Flavonol content was determined by a colorimetric method based on the complexation of flavonols with aluminum chloride in the presence of sodium acetate, forming a stable complex with maximum absorbance at 440 nm [21]. Quantification was carried out using quercetin as the external standard, and measurements are expressed as milligrams of quercetin equivalents per gram of NSBE (mg QE/g). Finally, total flavonoid content was determined by an aluminum chloride colorimetric assay based on the formation of a stable flavonoid–Al^3+^ complex under alkaline conditions. Flavonoids interact with aluminum ions via hydroxyl groups on the A and C rings, producing a chromogenic complex whose absorbance, measured at 510 nm, is proportional to flavonoid concentration. Quantification was evaluated using a (+)-catechin calibration curve, and results were expressed as milligrams of catechin equivalents per gram of NSBE (mg CTE/g) [22]. The biological assays were performed using the final extract normalized by gallic acid equivalents (GAE).

### 2.3. Cell Lines, Culture Conditions, and Treatment Schedule

HUVEC (normal primary human umbilical vein endothelial cells), HCT-8 (human ileocecal adenocarcinoma cells), and A549 (lung adenocarcinoma epithelial cells) cells were obtained from the Rio de Janeiro Cell Bank (Rio de Janeiro, Brazil). The cell cultures were grown in Dulbecco’s Modified Eagle Medium/Nutrient Mixture F-12 Ham (DMEM/F-12) supplemented with 1% penicillin-streptomycin solution (100 U/mL penicillin and 0.1 mg/mL streptomycin) and 10% fetal bovine serum (FBS), at 37 °C in a humidified atmosphere containing 5% CO_2_.

The NSBE stock solution (1000 µg GAE/mL) was prepared in ultrapure water. For the experimental assays, the corresponding working concentrations were obtained by diluting the stock solution in culture medium. The cisplatin (3.33 mM) and artesunate (0.016 mM) stock solutions were solubilized in NaCl 0.9% and absolute ethanol, respectively, and the working concentrations were diluted in culture medium.

### 2.4. Effect of NSBE on Cell Adhesion

The adhesion capacity of A549 and HCT-8 cells was assessed following a 1 h pre-treatment with NSBE in cell suspension at concentrations ranging from 10 to 50 μg GAE/mL. Treated cells were then transferred to 96-well plates (5 × 10^4^ cells per well) and incubated for 24 h at 37 °C under 5% CO_2_ in the continued presence of NSBE. To prevent detachment of adherent cells, a gentle washing step with PBS was performed to remove non-adherent cells. Subsequently, the adherent cells were stained with crystal violet (0.5% *v*/*v*) for 30 min, then solubilized with SDS (1% *w*/*v*) at 37 °C for 30 min. The absorbance was measured at 570 nm using a microplate reader (Synergy TM 234 H1, Biotek Instruments, Agilent, Winooski, VT, USA). Outcome measures were normalized using the control culture as the reference [23].

### 2.5. Antiproliferative Activity of NSBE

The clonogenic assay, also known as the colony formation assay, was used to assess the inhibitory effects of NSBE on proliferation and colony-forming ability in A549 and HCT-8 cell lines [24]. Cells were seeded into 6-well plates at a density of 3 × 10^3^ cells per well and incubated at 37 °C in a humidified atmosphere of 5% CO_2_ for 24 h to allow proper cell attachment. Subsequently, the culture medium was replaced with fresh medium supplemented with 2% fetal bovine serum (FBS) and NSBE at concentrations of 2.5 and 5 μg GAE/mL. Cells were exposed to the treatments for 24 h, followed by incubation for an additional 6 days in a growth medium containing 2% FBS, which was refreshed every two days. On day 7, the colonies were fixed with methanol for 30 min and stained with 0.5% crystal violet (*w*/*v*) for 30 min. Colony quantification was performed using ImageJ software, and the results were expressed as a percentage relative to the control group [25].

### 2.6. Antimetastatic Activity of NSBE Through Migration by Wound Healing Assay

Cell migration was evaluated using an adapted scratch assay with minor modifications. For this, HCT-8, and A549 cell lines were seeded into a 24-well plate at 3 × 10^5^ cells/well. After 24 h of adhesion, mechanical disruption of the cell monolayer was performed with a sterile micropipette tip, after which detached cells were removed by two washes with PBS. The adhered cells were treated with culture medium with 2% FBS (negative control) or NSBE at different concentrations (5 to 20 µg GAE/mL) diluted in the same culture medium. Image of the wounded regions was performed at 0, 24, and 48 h using an inverted microscope equipped with a camera (10× magnification; Zeiss Primovert, Zeiss, Oberkochen, Germany). The extent of cell migration was quantified as the percentage of wound closure relative to the control group, based on temporal changes in wound area measured with ImageJ software (version 1.53i; National Institutes of Health, Bethesda, MD, USA). To limit the influence of cell proliferation on migration measurements, assays were conducted under low-serum conditions (2% FBS). At the concentrations tested, NSBE did not significantly affect cell viability, as verified by MTT assays [26].

### 2.7. Evaluation of the Genotoxicity of NSBE by Chromosomal Aberrations Assay

The chromosomal aberration assay was performed to evaluate whether the NSBE has protective activity against chromosomal damage or if it induces genotoxicity. A549 cell line was selected for stable adherence and a well-characterized cell-cycle profile [27]. Cells were initially seeded in 25 cm^2^ flasks at an initial concentration of 5 × 10^5^ cells per flask. For the positive control, cells were exposed to 4 μM cisplatin, known for its ability to damage DNA, while the negative control received only the culture medium. The treatment groups received isolated concentrations of NSBE (10, 25, and 50 μg GAE/mL) and also these same concentrations in combination with 4 μM cisplatin. After incubation at 37 °C for 48 h, 200 μL of 0.0016% colchicine (Sigma Aldrich, São Paulo, Brazil) solution, an antimitotic known to prevent metaphase, was added to each group, and the flasks were incubated for an additional 6 h. The cells were then incubated in a hypotonic 75 mM KCl solution for 10 min at 37 °C, which promotes chromosome dispersion. They were then fixed and stained with Giemsa stain. Chromosomal aberrations were evaluated according to established breakage criteria, and the aberration rate (%) was determined by the percentage of chromosomal breaks in relation to the total number of chromosomes analyzed [28].

### 2.8. In Vitro Antimalarial Properties

The antiplasmodial activity of NSBE was evaluated in vitro against the 3D7 (chloroquine-sensitive) and W2 (chloroquine-resistant) strains of *Plasmodium falciparum*. The strains were cultivated in RPMI 1640 medium (Gibco, New York, NY, USA) supplemented with 10% Albumax II (Gibco, New York, NY, USA) and 4% hematocrit (Erythrocytes, type O^+^). Incubation was carried out at 37 °C using the candle jar method, with culture medium replacement every 48 h. Parasitemia was monitored through Panoptic fast-stained blood smear analysis (Renylab, Barbacena, MG, Brazil) [29].

After synchronization with 5% sorbitol (Sigma Aldrich, St. Louis, MO, USA) to obtain ring-stage parasites, the antiplasmodial assay was conducted in 96-well microplates using serial dilutions of NSBE ranging from 40 to 0.31 µg GAE/mL (1:1 *v*/*v*). Each well received the parasitized erythrocyte suspension prepared in RPMI 1640 medium containing 2% hematocrit and 1% parasitemia. Non-parasitized erythrocytes (2% hematocrit) were used as negative controls, while untreated infected cultures served as positive controls. Following 48 h of incubation, a lysis buffer solution containing 0.1 µL/mL SYBR Gold nucleic acid gel stain (Sigma Aldrich, São Paulo, Brazil) was added to each well to lyse the erythrocytes and stain parasite DNA. The fluorescence intensity was measured at 485 nm (excitation) and 520 nm (emission) [4]. Following the viability assay with the W2 and 3D7 strains, parasitized cultures at 2% hematocrit and 1% parasitemia were treated with the respective IC_50_ concentrations of the NSBE, and images were acquired at 0, 8, 24, and 48 h to determine the effect of the extract on the intraerythrocytic cycle of the parasite.

To assess whether the NSBE could potentiate the antiplasmodial effect of artesunate, combination assays were conducted using *P. falciparum* 3D7 and W2 strains. The IC_50_ of artesunate was determined in the absence and presence of fixed concentrations (1 or 0.5 µg GAE/mL) of NSBE. Artesunate stock solutions were prepared in absolute ethanol. Working concentrations were obtained by dilution in culture medium, and the final ethanol concentration did not exceed 0.2% (*v*/*v*). Vehicle control groups containing the same ethanol concentration were also included.

Synchronized ring-stage parasites were exposed to serial dilutions of artesunate together with a constant concentration of the extracts for 48 h. Parasite proliferation was quantified using a lysis buffer solution containing the SYBR Gold fluorescence method. The IC_50_ values obtained for artesunate alone and in combination were compared to determine potentiation or additive effects [30].

To compare cytotoxic effects in *P. falciparum* and cell cultures, cell viability was determined using the MTT assay (3-(4,5-dimethylthiazol-2-yl)-2,5-diphenyltetrazolium bromide), according to the protocol described by [16]. The selectivity index (SI) was calculated as the ratio between the IC_50_ values obtained for HUVEC cells in this study and those determined for the 3D7 and W2 strains of *P. falciparum* [31].

### 2.9. Statistical Analysis

All experiments were performed in quadruplicate, and results are expressed as mean ± standard deviation (SD). Cell adhesion, migration and colony formation assays were analyzed by one-way analysis of variance (ANOVA), followed by Tukey’s post hoc test for multiple comparisons. Chromosomal aberrations were evaluated using the chi-square test. Dose–response curves for antiplasmodial activity were obtained by non-linear regression analysis, with the goodness of fit assessed by the coefficient of determination (R^2^). Statistical significance was considered at *p* < 0.05 and *p* < 0.01. All statistical analyses and graphical representations were performed using GraphPad Prism^®^ software (version 8.0, USA).

## 3. Results and Discussion

### 3.1. Phenolic Composition and Chemical Characterization of NSBE

The chemical composition of the NSBE reveals a complex matrix enriched in structurally diverse phenolic constituents. In this study, we quantified key phenolic subclasses, including ortho-diphenolics, condensed tannins, flavonols, and total flavonoid content (Table 1). This profile is consistent with the broader phytochemical composition reported for coniferous species, in which tannins, stilbenes, phenolic acids, and multiple flavonoid classes are commonly identified [30]. Moreover, these findings complement previous analyses performed with the same batch of this NSBE by our research group, which demonstrated that NSBE contains a galactoglucomannan (GGM)-rich matrix together with a defined pool of phenolic compounds, dominated by epicatechin (≈177 µg/g) and catechin (≈45 µg/g), which were quantified by high-performance liquid chromatography (HPLC) [7], as well as a total reducing phenolic content of ~33 mg GAE/g [3,7]. Previous studies employing pressurized hot water extraction in forestry by-products, such as sawdust from commercially exploited tree species including *Pinus pinaster* and *Betula pendula*, have reported high total phenolic contents (121.3 and 187.43 mg GAE/g, respectively) [3,32]. Comparable values have also been reported for aqueous bark extracts of *Picea abies* and *Fagus sylvatica* obtained by ultrasound-assisted extraction, which exhibited total phenolic contents of 84.2 and 40.6 mg GAE/g, respectively [33] Phenolic levels vary according to extraction parameters, including the extraction method, solvent-to-matrix ratio, type of matrix, extraction time, temperature, and solvent used [34]. In pressurized hot water extraction (PHWE) at subcritical temperatures (160–180 °C), hemicelluloses such as galactoglucomannan can also be extracted. Increasing temperature promotes hemicellulose hydrolysis and reduces the dielectric constant of water, enabling the solubilization of more semi-polar compounds and improving water penetration into the sample matrix due to decreased viscosity [3,17]. In this context, the co-extraction of polysaccharides and free phenolics within the same matrix may contribute to the chemical stability, compositional integrity, and modulation of the extract’s bioactivity [35,36].

### 3.2. NSBE Impaired Cell Adhesion

**Figure 1:**
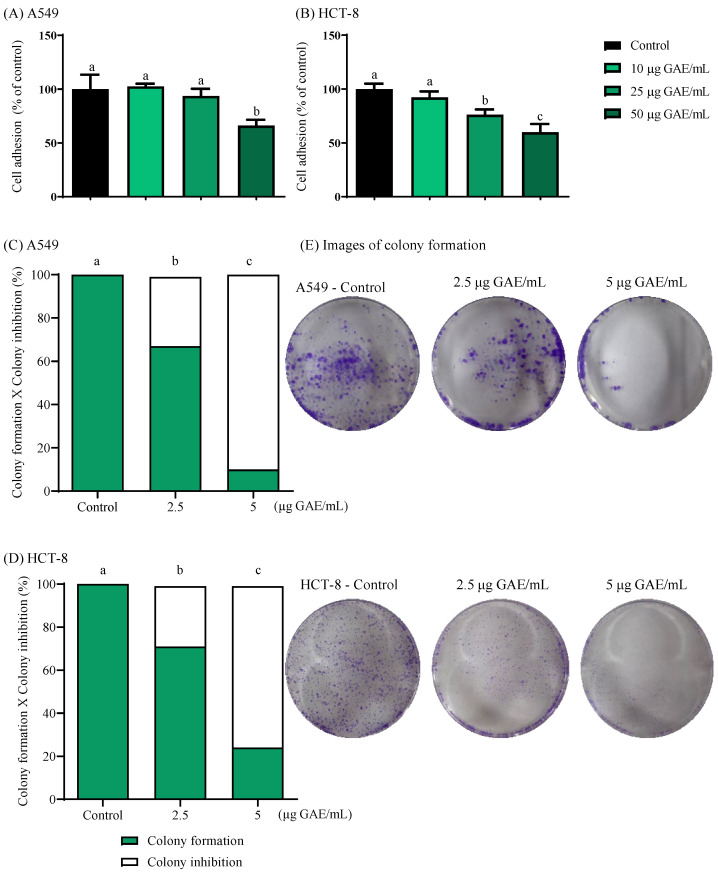
Cell culture overview of NSBE influence. Effects of NSBE at concentrations ranging from 10 to 50 µg/mL on cell adhesion (**A**,**B**) and from 2.5 to 5 µg/mL on colony formation (**C**,**D**). Representative well images for each treatment are shown in (**E**). Statistically significant differences among groups are indicated by different letters (a–c), as determined by one-way ANOVA followed by Tukey’s post hoc test (*p* < 0.05).

Adhesion and migration are essential steps in the metastatic cascade, enabling tumor cells to detach from the primary site and colonize distant tissues [23]. In the present study, treatment with NSBE was associated with a gradual attenuation of tumor cell adhesion in response to its increasing concentrations. In A549 cells, adhesion decreased by 33.96% at 50 µg GAE/mL, while in HCT-8 cells, reductions of 23.76% and 40.15% were observed at 25 and 50 µg GAE/mL, respectively (Figure 1), indicating that the phenolic constituents of NSBE interfere with cell–substrate interactions relevant to tumor progression. Importantly, the concentrations of NSBE used in the adhesion assays are based on the IC_50_ values reported for A549 and HCT-8 cells in previous toxicological evaluations of the same extract, indicating that the observed anti-adhesive effects are not attributable to cytotoxicity but rather to specific interference with cell–substrate interactions [7].

Furthermore, tumor cells inherently display a greater capacity for adhesion due to the overexpression and hyperactivation of adhesion molecules, particularly integrins, which are essential mediators of cell-extracellular matrix adhesion, coupling extracellular ligands to the actin cytoskeleton and coordinating focal adhesion assembly, processes critical for migration and metastatic dissemination [36,37]. In this context, the marked reduction in adhesion observed in A549 and HCT-8 cells suggests that NSBE may interfere with integrin-mediated interactions and the stability of focal adhesion complexes. 

Polyphenols compromise focal adhesion dynamics and integrin-dependent interactions, ultimately attenuating tumor cell migration and invasiveness [38]. Epicatechin, a phenolic constituent of NSBE, reinforces this mechanism by modulating the FAK/Src and PI3K/Akt pathways, suppressing MMP-9 expression, and upregulating key metastasis-suppressor genes such as CDH1, PTEN, and BRMS1 [39]. Collectively, these molecular events impair cytoskeletal reorganization, extracellular matrix degradation, and cellular motility. Moreover, the anti-adhesive properties observed for NSBE likely arise from the synergistic actions of its phenolic profile, particularly epicatechin, highlighting its potential to disrupt early molecular events involved in metastatic progression.

### 3.3. NSBE Inhibits the Colony Formation

Proliferation dynamics of A549 and HCT-8 cancer cells following NSBE treatment were assessed using the clonogenic assay. Unlike conventional viability-based cytotoxicity assays, which predominantly capture metabolic inhibition after continuous exposure of cancer cells to the cytotoxic agent, the clonogenic assay evaluates the long-term proliferative potential of tumor cells after treatment withdrawal. This distinction is critical, as many cytotoxic agents induce transient cell-cycle arrest without immediately compromising cell survival, allowing cells to recover once the treatment is removed. By incorporating a 7-day extract-free recovery period, the clonogenic assay provides a measure of true tumor-cell killing and the ability of surviving cells to sustain proliferation [40]. Thus, under these conditions, NSBE demonstrated the ability to reduce the clonogenic potential of both cancer cell lines, exhibiting a dose-dependent antiproliferative effect that reduced colony formation relative to the control by 32% and 90% in A549 cells and by 28% and 75% in HCT-8 cells at 2.5 and 5 µg GAE/mL, respectively (Figure 1).

The anti-clonogenic effect observed may be closely associated with the ability of the phenolic constituents of NSBE to modulate signaling pathways related to cell-cycle progression and cell survival, which are key mechanisms through which plant extracts inhibit cancer cell proliferation [41,42]. In particular, catechin, one of the major phenolics in NSBE, has been reported to influence cell-cycle control and survival pathways in A549 cells, by upregulating the CDK inhibitor p21, suppressing Cyclin E1 and AKT/p-AKT in A549 cells [43]. This is consistent with the nearly 100% reduction in colony formation observed in A549 cells at the 5 µg GAE/mL concentration.

In this sense, the diverse bioactive composition of NSBE may promote synergistic effects by simultaneously influencing several signaling pathways involved in tumor growth and progression, often with higher efficacy than individual compounds [44]. Indeed, condensed tannins may act in concert with other phenolic constituents present in the extract, modulating markers associated with apoptosis and cell-cycle control, such as Bax, Bcl-2, and the JAK2/STAT3 signaling pathway [45]. Thus, the intrinsic phytochemical complexity of the phytocomplex may contribute to the antiproliferative potential observed for NSBE.

### 3.4. Migration Profile of A549 and HCT-8 Cells Treated with NSBE

Collective cell migration plays a central role in tissue morphogenesis and wound closure, functioning alongside the well-known single-cell migration used in processes such as immune cell trafficking. In the context of wound-healing assays, epithelial or fibroblast monolayers migrate directionally toward the scratch gap, driven by leader cells at the wound edge and supported by follower cells that maintain structural integrity [33].

**Figure 2:**
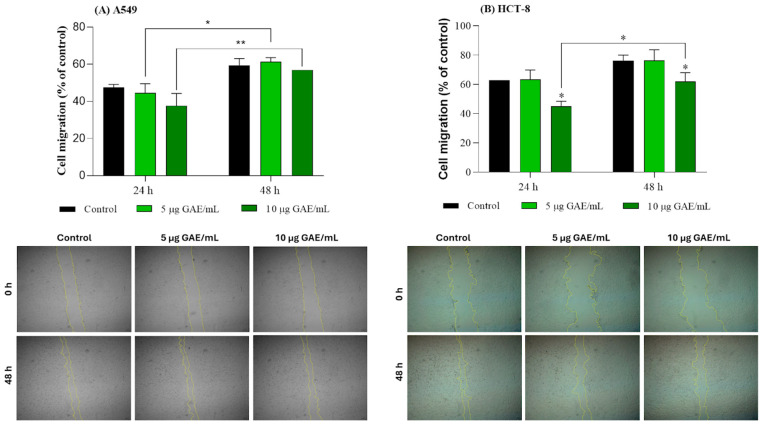
Migration of A549 (**A**) and HCT-8 (**B**) cells treated with NSBE (5–10 µL GAE/mL) for 24 and 48 h. Differences among groups were analyzed by one-way analysis of variance (ANOVA), followed by Tukey’s post hoc test. * *p* < 0.05 and ** *p* < 0.01 indicates statistically significant differences compared with the control group or between 24 h and 48 h within the same treatment concentration, as indicated.

Cell migration assessed by the wound healing assay showed divergent responses to NSBE in A549 and HCT-8 cells (Figure 2). For A549 cells, comparable migration patterns were observed in control and treated conditions at both time points, with the limited increase detected at 48 h attributable to the natural progression of wound closure over time. Microscopic images supported these findings, as the reduction in the wound area over time occurred similarly across all conditions. A different response has been reported, in which a 24 h treatment with an ultrasound-assisted aqueous extract obtained from *Picea abies* bark reduced A549 cell migration compared with the negative control [46]. Differences in the plant material used (bark versus sawdust), extraction methodology, extract composition, and dosage may account for the discrepancies observed between studies.

In contrast, HCT-8 cells exhibited sensitivity to the extract, with a 24 h treatment of 10 µg GAE/mL already resulting in a reduction in migration compared with the control. This inhibitory effect persisted after 48 h, although its proportion was smaller, indicating that the cells were able to migrate. The micrographs corroborated the quantitative data, showing visibly delayed wound closure in the treated groups relative to the control.

The coordinated movement of cell migration is facilitated by intercellular junctions that remain preserved, allowing neighboring cells to stay physically connected and to transmit mechanical forces across the tissue. Adherens junctions, primarily mediated by homotypic cadherin interactions, are essential for this behavior, ensuring that the advancing front moves as an integrated sheet to progressively close the wound space [33]. The impact on the cell migration process by wound healing can occur through several mechanisms: (i) modulation of adhesion molecules such as αvβ3 and β1, negatively affecting the expression or activity of integrins, reducing the tumor cell’s ability to adhere to the ECM and consequently decreasing the driving force for migration; (ii) interference in signaling pathways essential for cell adhesion and migration (PI3K/Akt), suppressing the cell motility necessary to fill the wound; (iii) impact on the cytoskeleton through modifications of actin (such as Rho GTPases proteins), compromising the dynamic reorganization of the cytoskeleton necessary for cell protrusion [33,36]. Therefore, the migration inhibition observed in HCT-8 cells may reflect this dysregulation of adhesive interactions and the signaling pathways associated with them, even though the A549 cells were not affected under the same circumstances. These results suggest a cell line–dependent response and a potential antimetastatic action in colorectal cancer cells.

The differential migration responses observed between A549 and HCT-8 cells may be rooted in distinct integrin-mediated adhesion and downstream signaling networks that vary between tumor types. Integrins act as bidirectional signaling receptors that link the extracellular matrix (ECM) to the actin cytoskeleton, initiating focal adhesion complexes and influencing cell motility through pathways such as FAK/Src and Rho GTPase activation, which are essential for cytoskeletal reorganization and migratory dynamics in cancer cells [47]. Recent evidence shows that integrin engagement not only regulates the assembly and disassembly of adhesion complexes but also modulates Rho family GTPase activity, thereby coordinating the balance between protrusion formation and contractility necessary for directed migration [47]. Furthermore, integrin signaling can vary with expression profiles and activation states of specific integrin subunits in different cancer cell lines, contributing to lineage-specific migratory behaviors during collective movement [48]. In this context, the selective impairment of migration in HCT-8 cells likely reflects their reliance on dynamic integrin-driven adhesion and cytoskeletal signaling for wound closure. In contrast, A549 cells may engage alternative or compensatory mechanisms less sensitive to the perturbations induced by NSBE, supporting a cell line–dependent response and suggesting a potential antimetastatic action in colorectal cancer models.

### 3.5. NSBE Protected Against Cisplatin-Induced Chromosomal Damage

**Figure 3:**
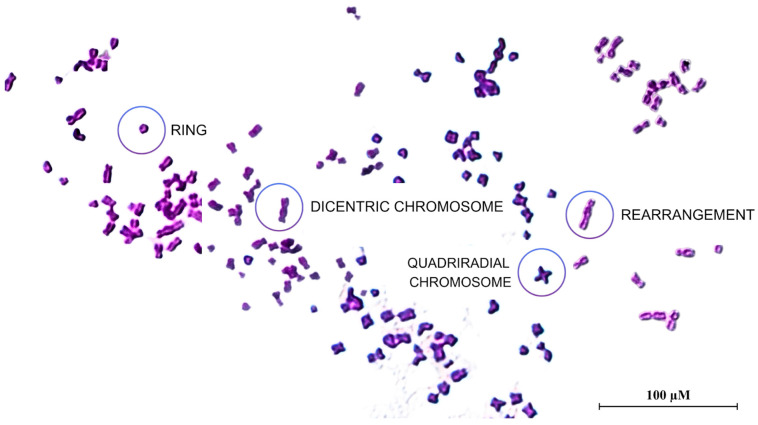
Metaphase spreads of A549 cells captured at 1000× magnification, highlighting chromosomal aberrations including ring chromosomes (R), dicentric chromosomes (DC), quadriradial chromosomes (QC), and structural rearrangements (RE).

To preserve genomic stability, cells must accurately replicate their DNA before each division and rely on efficient mechanisms to detect, signal, and repair DNA damage [49]. Chromosomal instability, a form of genomic instability observed in cancer and congenital disorders, is commonly associated with the action of mutagens and characterized by numerical or structural chromosomal alterations, such as rings, dicentric and quadriradial chromosomes, and structural rearrangements [50], as illustrated in the representative metaphase spreads shown in Figure 3. In this context, plant extracts have long been recognized for their therapeutic potential; however, evidence also indicates that some extracts may exert toxic, genotoxic, or mutagenic effects. Accordingly, a comprehensive assessment of their genotoxic profile is essential to ensure safety and guide their rational application [51].

Building on this premise, the present study evaluated the potential protective effect of NSBE against cisplatin-induced chromosomal damage in A549 cells. The extract did not induce chromosomal damage in cells exposed only to NSBE, indicating that it is not genotoxic under the tested conditions. Moreover, when combined with cisplatin, NSBE at 10 µg GAE/mL reduced the frequency of chromosomal aberrations from 1.41% in the cisplatin-treated positive control to 0.88%, indicating a protective effect on DNA integrity (Table 2).

We hypothesize that this response is consistent with the extract’s composition, particularly its catechin and epicatechin content [3,7], which is known to modulate cellular signaling pathways such as the MAPK/ERK pathway. Inhibition of this pathway has been associated with reduced oxidative stress; these compounds can mitigate genotoxic damage and preserve genomic stability through their hydroxyl-rich structure, which efficiently scavenges reactive oxygen species. Such mechanisms may have contributed to maintaining chromosomal integrity and, consequently, minimizing the genotoxic effects induced by the chemotherapeutic agent [52,53].

Additionally, mannose present in the NSBE extract [7] can hinder the efficient utilization of glucose, the preferred energy and biosynthetic substrate in cancer cells, a metabolic disruption that has been associated with increased cellular sensitivity to DNA-damaging chemotherapeutic agents, such as cisplatin, thereby potentially enhancing the cellular response to this drug [54]. Taken together, the protective, and sensitizing properties of NSBE suggest that this extract may modulate key cellular pathways to preserve genomic stability during cisplatin treatment, highlighting its potential value in therapeutic contexts.

Beyond polyphenol-driven redox modulation, previous compositional analyses of NSBE support a secondary hypothesis involving the presence of mannose within the GGM-rich matrix, which may influence biological responses through carbohydrate-mediated mechanisms [7]. Mannose may interfere with the efficient utilization of glucose, the primary energy and biosynthetic substrate in cancer cells, thereby inducing a metabolic perturbation that has been associated with increased cellular sensitivity to DNA-damaging chemotherapeutic agents, such as cisplatin, potentially enhancing the cellular response to this drug [55]. Taken together, while distinguishing between experimentally supported findings and hypothesis-driven interpretations, the protective and sensitizing properties attributed to NSBE suggest that this extract may modulate key cellular pathways involved in the preservation of genomic stability during cisplatin treatment, highlighting its potential relevance in therapeutic contexts.

### 3.6. NSBE Exerts Antimalarial Activity in 3D7 and W2 Strains

**Figure 4:**
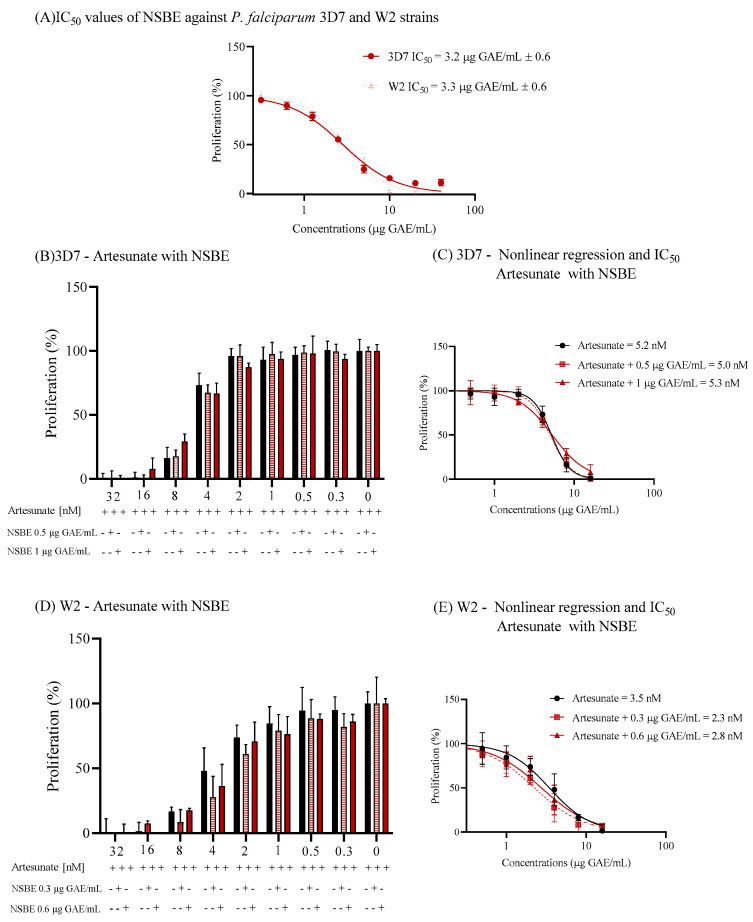
In vitro assessment of Norway spruce by-product extract (NSBE) as an antiplasmodial agent against *Plasmodium falciparum* 3D7 and W2 strains and in combination with artesunate (**A**) NSBE dose–response profiles in chloroquine-sensitive (3D7) and chloroquine-resistant (W2) strains after 48 h of incubation, showing IC_50_ values of 3.2 ± 0.6 and 3.3 ± 0.6 µg GAE/mL, respectively. Parasite proliferation was quantified by SYBR Gold fluorescence. (**B**,**D**) Effect of NSBE (0.5 or 1.0 µg GAE/mL, fixed concentrations) on artesunate dose–response curves in 3D7 (**B**) and W2 (**D**) strains. (**C**,**E**) Nonlinear regression analysis, performed using the least-squares method, of artesunate IC_50_ values in the absence and presence of NSBE in 3D7 (**C**) and W2 (**E**), showing no significant shifts in potency and indicating a lack of synergistic or antagonistic interaction under the conditions tested.

The antiplasmodial activity of NSBE was examined in *Plasmodium falciparum* strains 3D7 and W2, which are sensitive and resistant to chloroquine, respectively. The extract exhibited potent inhibition of parasite proliferation, characterized by IC_50_ values of 3.2 ± 0.6 and 3.3 ± 0.6 µg GAE/mL for 3D7 and W2, respectively (Figure 4). These nearly identical IC_50_ values indicate that NSBE’s inhibitory activity is not influenced by chloroquine resistance and lies within the range typically considered promising for crude plant extracts in antimalarial screening.

Combination assays with artesunate demonstrated that NSBE does not alter the efficacy of this front-line antimalarial. In both *P. falciparum* strains, the IC_50_ values of artesunate varied by less than 10% in the presence of fixed NSBE concentrations (0.5 or 1.0 µg GAE/mL) compared with artesunate alone, with overlapping confidence intervals and superimposable dose–response curves (Figure 4). This lack of a meaningful shift in artesunate potency indicates the absence of synergistic or antagonistic interaction, suggesting that NSBE and artesunate act through independent or non-interacting mechanisms under the tested conditions.

From a therapeutic positioning perspective, the absence of interaction between NSBE and artesunate should not be interpreted as a limitation of the extract. On the contrary, this profile supports the compatibility of NSBE with front-line antimalarial therapy, indicating that its bioactivity does not interfere with the efficacy of a clinically established drug. This characteristic is particularly relevant in the context of functional ingredients, where concomitant use with standard pharmacological treatments is likely [56].

Rather than acting as a drug-modifying or potentiating agent, NSBE may be better positioned as a complementary bioactive extract with intrinsic antiplasmodial activity that can be safely co-administered alongside conventional antimalarial regimens. In this context, the lack of synergistic or antagonistic interaction reinforces the potential biomedical relevance of NSBE as a multifunctional bioactive ingredient, while minimizing concerns related to adverse drug–extract interactions [57]. This type of non-interfering profile has been reported for several plant-derived bioactive extracts evaluated alongside antimalarial therapies, where compatibility and safety during co-administration are considered advantageous over direct pharmacological potentiation [58,59].

Literature consistently shows that extracts with higher phenolic abundance tend to exhibit stronger antiplasmodial activity, both in chloroquine-sensitive and -resistant strains [59]. Given this context, flavanols such as catechin and epicatechin, together with tannins and other phenolic subclasses typically associated with NSBE, likely contribute to the observed activity [54,60]. Polyphenol-rich extracts and structurally related compounds have been reported to inhibit *P. falciparum* proliferation, supporting the hypothesis that the phenolics, not the polysaccharidic backbone, are the main driver of the antiplasmodial effect [54].

Still, it is improbable that a single molecule fully explains the IC_50_ values observed. Plant extracts often behave as phytocomplexes, in which different constituents act additively or synergistically on multiple parasite processes, including redox balance, membrane integrity, and metabolic pathways [61]. Mechanistically, these observations raise the hypothesis that part of NSBE activity could involve heme-related processes. Several flavonoids and polymethoxylated flavones can bind free heme and inhibit hemozoin (β-hematin) formation—an established antimalarial mechanism also targeted by quinoline drugs [62,63]. Notably, highly methoxylated flavonoids have been shown to combine strong heme binding with inhibition of intraerythrocytic hemozoin formation, correlating with antiplasmodial potency [64]. At the same time, some flavonoids display antiplasmodial effects without affecting hemozoin, indicating additional non-heme targets [63]. In light of these effects, heme binding or hemozoin formation may be considered as generating these hypotheses and justifying future targeted assays. In NSBE, the coexistence of epicatechin, catechin, and other redox-active phenolics within compounds from sawdust matrix may improve stability, uptake, or interaction with parasite structures, collectively driving growth inhibition in the low concentration range [64,65].

The lack of artesunate interaction reinforces a non-overlapping, broad cytostatic/cytotoxic mode of action rather than a single stage-specific target. The selectivity index (SI ≈ 13), calculated from the IC_50_ in HUVEC cells (43 µg GAE/mL), further highlights the preferential action of NSBE on the parasite relative to mammalian cells, surpassing the SI ≥ 10 threshold commonly considered suitable for antimalarial candidates [66].

### 3.7. Microscopic Evaluation of the Intraerythrocytic Cycle

**Figure 5:**
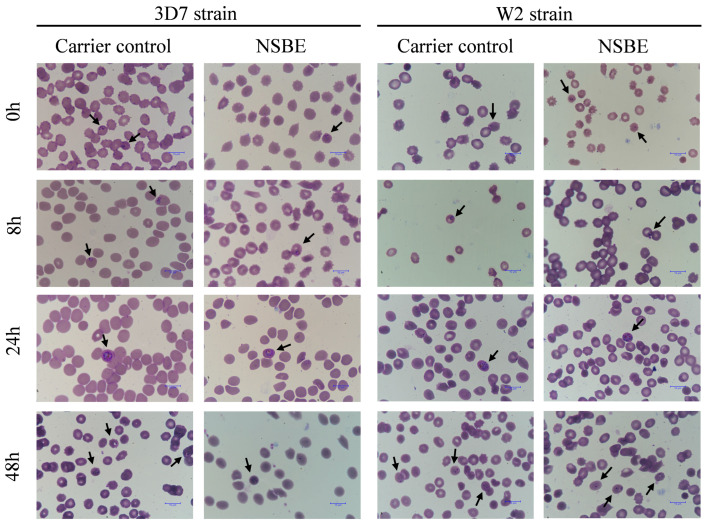
Microscopy images of synchronized *P. falciparum* 3D7 and W2 strains at different time points, stained using the rapid panoptic method. Smears show parasites cultured under control conditions (RPMI medium containing 10% Albumax II) and treated with NSBE at the IC_50_ values for each strain (3D7: 3.2 µg GAE/mL; W2: 3.3 µg GAE/mL). Arrows indicate intraerythrocytic structures.

Microscopic analysis of Giemsa-stained thin smears at 0, 8, 24, and 48 h showed no morphological abnormalities or evidence of stage-specific arrest in parasites exposed to NSBE (Figure 5). Rings, trophozoites, and schizonts preserved their typical morphology and progressed normally through the erythrocytic cycle when compared with untreated controls. These findings indicate that NSBE does not induce clear morphological alterations detectable by light microscopy, suggesting that its antiplasmodial activity is more likely driven by metabolic or redox-related perturbations than by interference with a discrete morphological transition.

In conclusion, NSBE inhibited the in vitro growth of chloroquine-sensitive and resistant *P. falciparum* strains without affecting intraerythrocytic progression or artesunate response.

## 4. Conclusions

The results presented herein underscore that the Norway spruce by-product extract (NSBE) exhibits relevant biological activities that support its potential use as a multifunctional functional food ingredient. The extract reduced adhesion and clonogenic capacity in A549 and HCT-8 tumor cells, indicating antiproliferative and antimetastatic effects. Although migration inhibition occurred only in HCT-8 cells, the response suggests a cell line–dependent sensitivity. NSBE did not induce chromosomal damage and showed a protective effect against cisplatin at 10 µg GAE/mL, reinforcing its toxicological safety under the tested conditions. Additionally, the extract displayed potent antiplasmodial activity in both chloroquine-sensitive and -resistant *Plasmodium falciparum* strains, with IC_50_ values below 3.5 µg GAE/mL and a selectivity index above the recommended threshold for natural antimalarial candidates.

Despite these promising findings, further in vivo validation is required to confirm the functional relevance, toxicological safety, and bioavailability of NSBE under physiological conditions. Such studies represent critical steps prior to its incorporation into food matrices and subsequent regulatory assessment as a functional food ingredient.

## Figures and Tables

**Table 1:** Phenolic matrix characterization and redox properties.

Chemical Compounds	Content
Ortho-diphenolics (mg CAE/g)	10.5 ± 0.2
Tannins (mg CTE/g)	2.7 ± 0.83
Flavonols (mg QE/g)	1.9 ± 0.2
Flavonoids (mg CTE/g)	16.4 ± 1.08
Epicatechin (µg/g) [5]	≈177
Catechin (µg/g) [5]	≈45
Total reducing phenolic content (mg GAE/g) [5]	~33

Note: Quantification of ortho-diphenolics expressed as mg chlorogenic acid equivalents per gram (mg CAE/g), tannins and flavonoids expressed as mg catechin equivalents per gram (mg CTE/g), flavonols expressed as mg quercetin equivalents per gram (mg QE/g), epicatechin and catechin expressed as µg/g, and total reducing phenolic content expressed as mg gallic acid equivalents per gram (mg GAE/g). Values are presented as mean ± standard deviation, when applicable.

**Table 2:** Chromosomal aberration analysis in A549 cells following in vitro treatment with NSBE.

NSBE	CIS	TC	Aberrant Type				TNCA	CA (%)	NSBE	CIS	TC	Aberrant Type				TNCA	CA (%)
			R	DC	QC	RE						R	DC	QC	RE		
NC	-	5072	1	11	6	31	49	0.97 ^a^	NC	-	5072	1	11	6	31	49	0.97 ^a^
PC	yes	5323	2	17	5	51	75	1.41 ^b^	PC	yes	5323	2	17	5	51	75	1.41 ^b^
0	-	5263	1	21	3	28	53	1.01 ^abc^	10	yes	5656	0	7	3	40	50	0.88 ^ac^
25	-	5288	2	1	1	40	44	0.83 ^ac^	25	yes	4935	2	4	1	44	51	1.03 ^abc^
50	-	4869	0	9	0	32	41	0.84 ^ac^	50	yes	4890	1	11	1	45	58	1.19 ^abc^

Note: NSBE: Norway Spruce By-product Extract (µg GAE/mL); CIS: cisplatin 4 μM; TC: total cells; R: ring; DC: dicentric chromosome; QC: quadriradial chromosome; RE: rearrangement; TNCA: number of cells exhibiting chromosomal alterations; CA: chromosomal aberration frequency; NC: negative control; PC: positive control. In the presence of multiple types of chromosomal aberrations during metaphase, the number of chromosomal aberrations was counted as 1. Letters a–c indicates significant differences (*p* < 0.05) according to Tukey’s test.

## Data Availability

The original contributions presented in this study are included in the article. Further inquiries can be directed to the corresponding authors.
